# Forensic Pathology and Legal Issues in COVID-19: Case Report and Literature Review

**DOI:** 10.7759/cureus.56807

**Published:** 2024-03-24

**Authors:** Maria J Kingsley-Godwin, Ivan I Tsranchev, Veselin Belovezhdov, Pavel Timonov, Antoaneta Fasova, Metodi Goshev, Biliana Mileva, Alexandar Alexandrov

**Affiliations:** 1 Department of Forensic Medicine and Deontology, Medical University of Plovdiv, Plovdiv, BGR; 2 Department of General and Clinical Pathology, Medical University of Plovdiv, Plovdiv, BGR; 3 Department of Anatomy, Histology and Embryology, Medical University of Plovdiv, Plovdiv, BGR; 4 Department of Forensic Medicine and Deontology, Medical University Sofia, Sofia, BGR

**Keywords:** omicron, forensic pathology, ards, pneumonia, autopsy, sars-cov-2, covid-19

## Abstract

Autopsy investigations of deaths following nosocomial coronavirus disease 2019 (COVID-19) infection have enormous medical and social significance, as autopsies are essential for the correct statistical recording of COVID-19 deaths, presenting new lessons, which is important to policymakers and in the improvement of public health in general.

Our study is based on the presentation of a case of a road traffic accident involving an elderly 73-year-old female with a complication of nosocomial COVID-19 infection and death leading to forensic pathological investigation. This involved autopsy, histopathological examinations, and other tests, and highlighting the importance of medicolegal matters, including the legal and ethical practicalities encountered in healthcare.

This article highlights the fact that patients who have sustained various traumatic injuries accompanied by nosocomial COVID-19 infection have higher risks of morbidity and mortality. The significance of the role of a Forensic Pathologist in dealing with the analysis of injuries and the performance of autopsies to determine the cause, mechanism, and manner of death. In addition, the important lesson of testing patients for COVID-19 more regularly during a long hospital admission period, to offer early treatment and isolation, including avoiding the further spread of the COVID-19 infection variants to patients and healthcare professionals, thereby minimising and preventing hospital-acquired infection and death is stressed.

## Introduction

A novel coronavirus was reported as the cause of a cluster of pneumonia cases in Wuhan, China at the end of 2019 [1]. Afterwards, the virus rapidly spread throughout the world, resulting in a global pandemic [1, 2]. Severe acute respiratory syndrome coronavirus 2 (SARS-CoV-2) was the name given to the virus, which causes coronavirus disease 2019 (COVID-19) [1, 2]. On 11 March 2020, the World Health Organization (WHO) declared COVID-19 “a global pandemic” [3]. 

In humans, the spectrum of COVID-19 ranges from asymptomatic infection to mild respiratory tract symptoms to severe pneumonia with acute respiratory distress syndrome (ARDS) and multiorgan dysfunction [1-4]. COVID-19 has been linked to various clinical conditions such as pharyngitis, conjunctivitis, other ocular involvements, Sjogren’s syndrome, and others. Hospital in-patients with long-term admission are at high risk of acquiring the infection. This is more complicated by emerging variants of COVID-19 from the ‘delta’ variant [5] to the recent ‘omicron’ variant, which has high transmissibility [6], and the current sub-variant, BA.2, which is rapidly spreading, and becoming predominant source of infections around the world [7-10]. Thus, our understanding of the spectrum of the disease as well as the optimal management strategies continues to evolve [8-10].

In addition, forensic pathologic findings could provoke serious expert challenges, especially when they involve trauma patients after road traffic accidents with the COVID-19 disease as a hospital-acquired infection. In this instance, the COVID-19 infection induces serious medicolegal problems in determining the causality between the initial injury, the subsequent COVID-19 infection, and the cause of the patient’s death. Thus, the purpose of this study is to analyze the role of forensic pathology and medico-legal issues in society, highlighting the importance of trends and issues in COVID-19 disease, and the importance of the legal and ethical practicalities encountered in healthcare, by presenting and discussing the issues in the case of a tragic road accident involving an elderly female patient, which was complicated by nosocomial COVID-19 infection that caused her death, leading to forensic pathological investigations to determine the nature and cause of death.

## Case presentation

A 73-year-old female was admitted to the Emergency Department of one of the university hospitals in Plovdiv, Bulgaria after a road traffic accident involving a truck rolling over both of her legs. The patient was admitted in severe clinical condition, in a state of traumatic shock with a blood pressure of 77/46 mmHg and a heart rate of 132 beats per minute (BPM). The results of the full blood count are as follows: hemoglobin of 82.0 G/L, red blood cell count of 3.03 10^12/L, hematocrit (HCT) of 0.263 l/l (Table 1). 

**Table 1:** Relevant clinical and laboratory patient parameters on admission

Parameters	Values	Reference Range/Normal Values
Blood pressure	77/46 mmHg	100-140/60-90 mmHg
Pulse	132/min	60-90/min
Haemoglobin	82.0 G/L	120-160 G/L
Red blood cells	3.03 10^12/L	3.9-5.3 10^12/L
Haematocrit (HCT)	0.263 L/L	0.36-0.47 L/L

On physical examination and during the diagnostic computed tomography (CT) scans, complete traumatic amputation of the left lower leg in the middle third of the tibia, huge traumatic tearing of the skin and soft tissues in the area of the right lower leg with fracture of the fibula, in combination with fractures of the 7th and 8th left ribs were established. Immediately after a complete surgical and orthopaedic clinical assessment, she was transferred to the Department of Orthopaedics and Traumatology. Her clinical condition was stabilized and she underwent surgical amputation of the left limb to the level of the knee joint. Intravenous antibiotic protective therapy and antiseptic treatment for both limbs were administered during the entire postoperative period. Routine Polymerase Chain Reaction (PCR) testing was performed for the SARS-CoV-2 virus on the first day of admission, and the result was negative. After two weeks of hospital treatment, a re-amputation was performed due to necrosis and inflammation of the operated left limb. In addition, a microbiological investigation of samples from the operative wound was performed and the results were negative. During this continual protective antibiotic treatment for about three weeks after the accident and admission period in the hospital, her body temperature increased to 39.4 °C with swelling, redness, warmth, and fluctuation over the right leg. As a result, surgical incisions and interventions were immediately performed in the area of inflammation, and antiseptic therapy was applied.

On the 27th day of her hospital stay, her condition gradually started to worsen with the presence of a cough and the development of bilateral exudative pleurisy. CT examination revealed the presence of bilateral inflammatory foci near the main bronchi and the periphery of the lungs. On the 31st day of the hospital stay, a new PCR test was performed and the result was positive for the SARS-CoV-2 virus. On the next day, she died with clinical manifestations of acute respiratory distress syndrome (ARDS) despite complete intensive resuscitation and oxygen therapy.

Following the death of the patient, the body was immediately transported to the Department of Forensic Medicine and Deontology at Medical University, Plovdiv, Bulgaria for routine forensic examination in order to determine the manner, the mechanism, and the cause of the death. Conventional forensic autopsy was performed with additional microscopic examination of cut sections of tissues, and postmortem virological testing for COVID-19.

**Figure 1:**
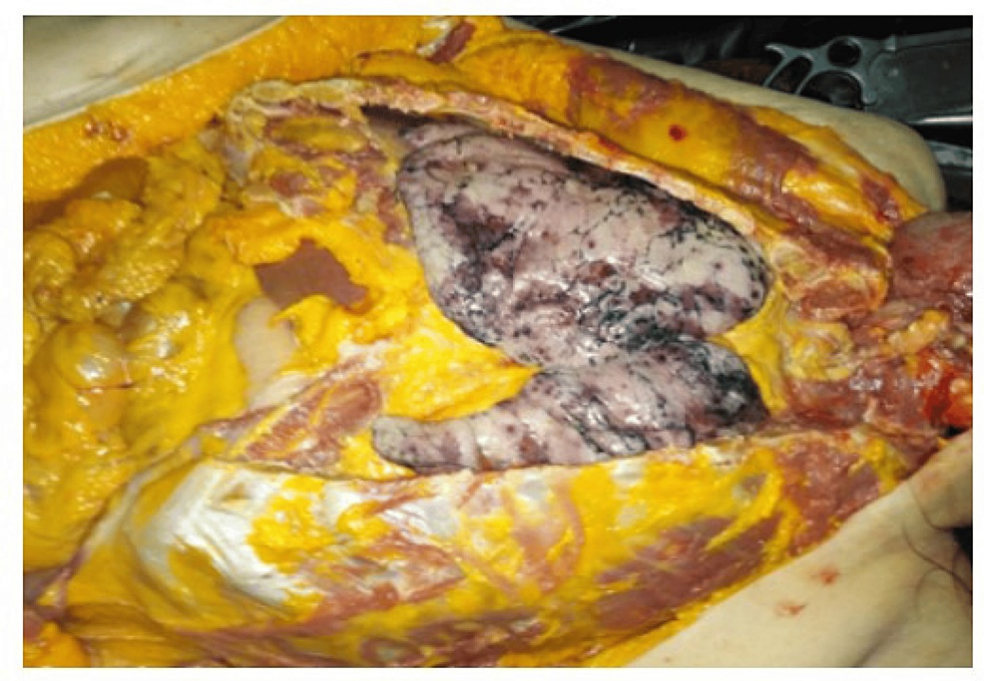
The parenchyma and the surface of the lungs ranging from pink to dark red in colour.

**Figure 2:**
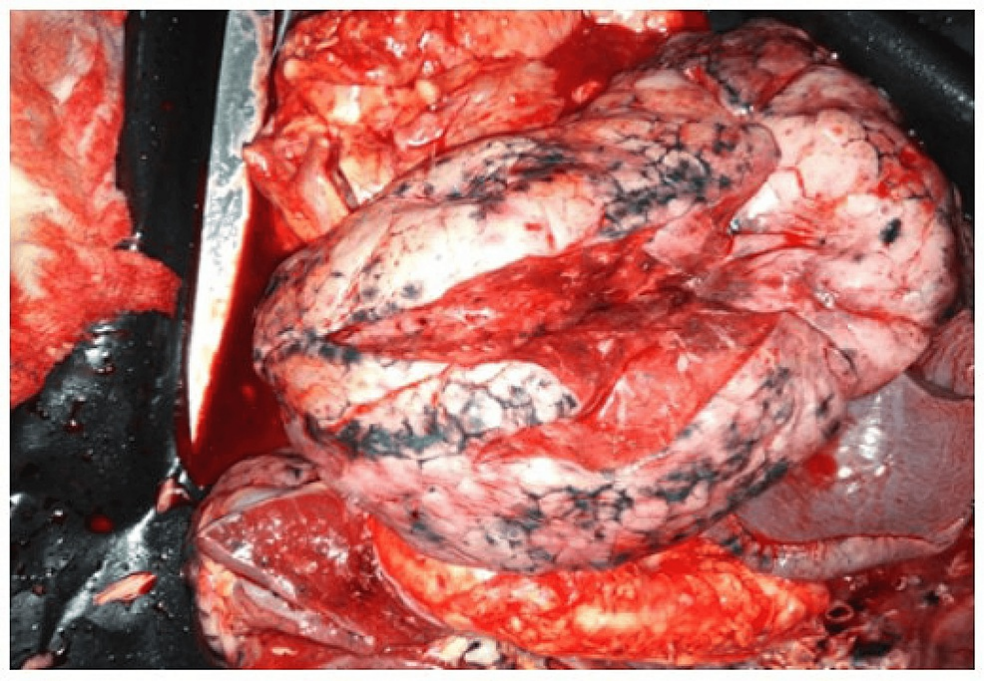
The parenchyma and the surface of the lungs with areas from pale pink to dark red in colour with irregular haemorrhagic areas.

On gross examination of the cranial cavity, there was severe cerebral oedema and well-manifested venous stasis. In addition, during the examination of the thoracic cavity, various features were seen, which included: accumulation of serous fluids in both intrapleural spaces; both lungs were congested, oedematous; the right lung weighed 660 grams and the left lung weighed 620 grams, with small petechial hemorrhages over their surface. Gross cut section of the lungs revealed diffusely firm and rubbery parenchyma with no palpable masses, and all lobes were affected equally. The bronchi were filled with frothy fluids and the surface of the lungs ranged from pale pink to dark red in colour with irregular haemorrhagic areas (as can be seen in Figure 1 and Figure 2). The hilar lymph nodes were slightly enlarged with a white-to-black cut surface.

The heart was normal externally and weighed 310 grams with mild atherosclerotic changes of the coronary arteries. Over the epicardium of the heart, pinpoint haemorrhages were found, and the blood within the major blood vessels was dark red. During the forensic examination of the abdomen, it was seen that the abdominal organs were severely congested: the liver weighed 1720 grams, the spleen weighed 320 grams, and the kidneys weighed 135 grams.

Organ samples were preserved for histological examination - they were fixed in 10% formalin solution for 24 hours, then histological samples with hematoxylin-eosin staining were prepared. Microscopic examination of cut sections of the brain revealed pericellular and perivasal oedema, including arteriolohyalinosis and lipofuscin staining that were seen in the cytoplasm of neurons. Microscopic examinations of the abdominal organs showed different findings, such as: acute venous stasis in the red pulp seen in the spleen; acute venous stasis present in the liver; and kidneys with hyperplastic medial proliferation into the small arteries, with arteriolohyalinosis, degenerative changes in tubular epithelial cells, and venous stasis.

On microscopy, the heart was hypertrophic, with interstitial fibrosis and lipomatosis, and the pancreas had interstitial fibrosis, lipomatosis, and degenerative changes. 

**Figure 3:**
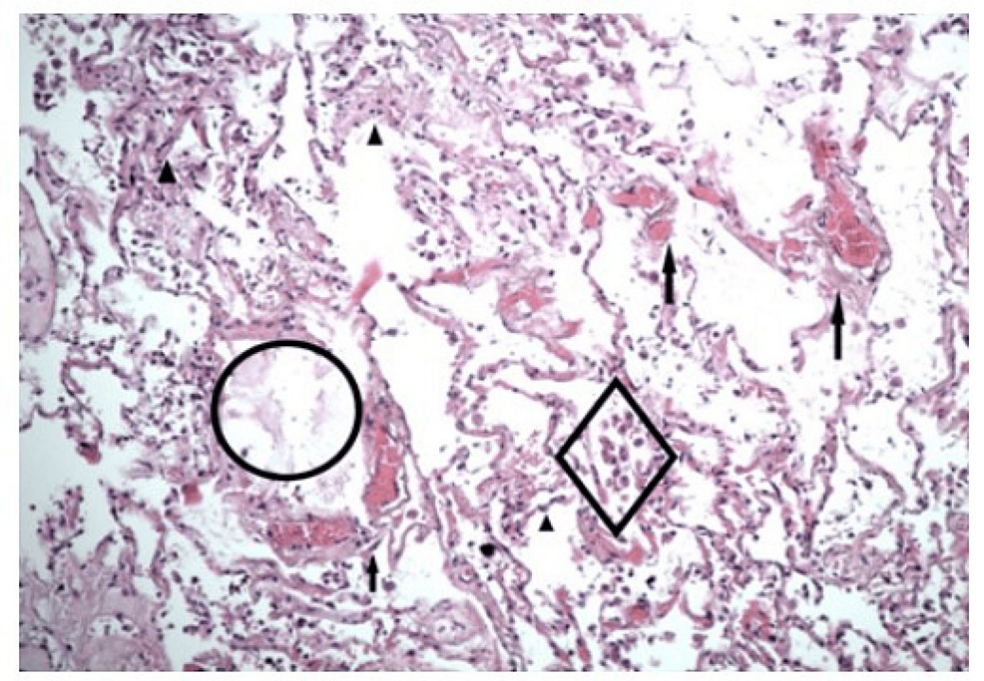
Hematoxylin-Eosin staining (zoom x200) of tissue samples from lungs showing interstitial inflammatory infiltrates (arrowheads), severe venous stasis (arrows), mild pulmonary oedema (circle), and macrophages inside the alveoli, some of them with hemosiderin (diamond).

In the samples obtained from the lungs for microscopic examination after hematoxylin-eosin staining, epithelial cells were seen with degenerative changes along with subepithelial lymphocytic inflammatory infiltrates (Figure 3). Furthermore, tissue samples of the lungs showed stasis of blood within the vessels, with interstitial infiltrates of lymphocytes and macrophages, and filling of the lung alveoli with macrophages, lymphocytes, erythrocytes, and siderophages, including the presence of partial atelectasis. 

## Discussion

In this article, we have analyzed the role of forensic pathology and medico-legal issues in society and highlighted trends and issues in COVID-19 disease, including the importance of medicolegal practicalities encountered in healthcare by presenting and discussing the issues in the case of a tragic road accident involving an elderly female patient, which was complicated by hospital-acquired COVID-19 infection, which caused her death. This gives rise to forensic pathological investigations to determine the nature and cause of death, including the need for an efficient virological testing strategy and preventive measures to control the spread of COVID-19 infection.

Historically, SARS has presented as a recurring public health challenge during the millennium - the first SARS epidemic in 2002 in China, the second major SARS epidemic in 2012 in the Middle East, and the current SARS pandemic, COVID-19/SARS-CoV-2 [10, 11]. The COVID-19 pandemic also raises new challenges for forensic pathologists. In this unprecedented situation, they must: determine the exact cause of death in cases with positive COVID-19 results, understand the pathological mechanisms leading to death in COVID-positive cases determine the manner of the death in such cases, and establish the relationship between proven COVID-19 infection, current trauma and the exact cause of death.

It has been stated in the literature that patients with concomitant traumatic injuries and COVID-19 infection have elevated risks of morbidity and mortality, especially in the age group of our case of people over 65 years of age [12]. Likewise, Karayiannis et al [13] in their study also demonstrated that the 30-day postoperative mortality rate in patients who are aged ≥ 65 is much higher in those who are COVID-positive versus those who are COVID-negative [14]. This is partially in line with our findings. However, our case has involved the use of the service of a forensic pathologist to determine the cause of death based on a detailed and complete autopsy and to confirm if the cause of death is in accordance with the manner of death observed and recorded clinically during the patient’s hospital admission period. This is essential as the patient had a negative PCR test for COVID-19 on the first day of admission with traumatic road traffic accident injuries, but this was complicated by the fact that she tested positive on the 31st day of admission after developing additional symptoms of pyrexia, cough with deteriorating symptoms, and died the next day, which raises many medico-legal issues.

As reported by Elder et al [14], there are four types of COVID-19 deaths: Type 1, with definitive COVID-19 death, where the cause of death is by autoptic pneumonia and/or ARDS; Type 2, with the probability of COVID-19 death, where the cause of death is by autoptic pneumonia and/or ARDS and different infectious etiology, for example, pulmonary embolism; Type 3, with the possibility of COVID-19 death of an equal alternative etiology, where the cause of death cannot be confirmed by postmortem, for example, arrhythmic issues in cardiomyopathy or pneumonia of other origins like exacerbated chronic obstructive pulmonary disease (COPD), aspirational pneumonia, etc.; and Type 4, with SARS-CoV-2 detection with etiology not related to COVID-19, and non-SARS-CoV-2 related cause of death such as acute myocardial infarction and complication of brain mass hemorrhage in hypertension.

Our case falls into Type 1 of the categories of SARS-CoV-2-positive deaths as stated by Elder et al [14]. Within this group, all cases with confirmed pneumonia or ARDS as the cause of death, with or without evidence of sepsis are included. As described in this paper, the patient we studied died from the clinical manifestation of ARDS as a result of nosocomial COVID-19 infection despite complete intensive resuscitation and oxygen therapy that was administered to her.

Furthermore, according to several scientific reports, the SARS-CoV-2 hospital-acquired infection rate is described as 12-15%, and hospital-acquired COVID-19 infection continues to be a very serious public health problem worldwide [15-20]. In our study, COVID-19 infection was established on the 31st day of hospital admission by performing a routine PCR examination [1, 20], while the first test performed on the first day during admittance was negative. The findings of our study give rise to serious expert problems in the process of our investigation because the relationship between events has a fundamental role in the determination of guilt in the committed illegal act, i.e., the road traffic accident. In addition, patients who have a combination of different traumatic injuries and acquired COVID-19 infection have higher risks for morbidity and mortality.

To the best of our knowledge, and evidence from the literature search, there is no previous analysis and in-depth descriptions of a tragic road accident with complication of nosocomial COVID-19 infection resulting in the death of an elderly female patient and involving the analysis of the role of forensic pathology in determining the nature of death, including medico-legal issues in it. In our study, the cause of death was determined as COVID-19-related pneumonia, confirmed by autopsy examination. This COVID-19-related death as confirmed in the histological studies in this case is the post-traumatic complication of the severe trauma resulting from the road traffic accident, and there is a well-established relationship between the initial injury and the fatal outcome that followed it [14-20].

Our case also indicates that medico-legal disputes focused on trauma patients with COVID-19 infection will increase in time and in such cases forensic pathologists must play their fundamental roles in contributing accurate data to physicians, public health agencies, police, and courts, thereby helping the criminal justice system. Consequently, we believe that there are lessons to be learnt from this case report, such as testing patients admitted to hospitals more frequently, putting isolation mechanisms in place in order to avoid the spreading of infections, and adhering to preventive measures like maintaining high-quality hygiene, like regular hand washing using sanitizer, etc., must be observed by staff and patients. These infection control mechanisms should be applicable in primary, secondary, and tertiary care settings, especially with the current rising infection rate with the latest omicron sub-variant of BA.2 in various parts of the world.

## Conclusions

In this article, we described a case of an elderly woman who had suffered severe injuries following a traumatic road accident, which was complicated by nosocomial COVID-19, leading to her death, and necessitated the services of forensic pathology due to the medicolegal legal issues that arose in the matter. As was also evident from the findings of the forensic investigations in this case, postmortems of the deceased with a confirmed COVID-19 infection can provide crucial data into the new coronavirus disease and its course. In addition, these findings will have significant implications for patients, their families, the judicial system, healthcare organisations and public health managers, and policymakers. In order to maintain high-quality healthcare for all populations in different communities, putting in place adequate testing strategies for SARS-CoV-2 for inpatients and maintaining preventive measures will lead to better health outcomes. Future work would involve the design and implementation of evidence-based guidelines for meeting these extremely important requirements in these unprecedented times with the currently evolving COVID-19 pandemic and its emerging new variants, as well as for the future.
